# Multiple-site decontamination regimen decreases acquired infection incidence in mechanically ventilated COVID-19 patients

**DOI:** 10.1186/s13613-022-01057-x

**Published:** 2022-09-02

**Authors:** Nicolas Massart, Florian Reizine, Pierre Fillatre, Philippe Seguin, Béatrice La Combe, Aurélien Frerou, Pierre-Yves Egreteau, Baptiste Hourmant, Pierre Kergoat, Julien Lorber, Jerome Souchard, Emmanuel Canet, Guillaume Rieul, Yannick Fedun, Agathe Delbove, Christophe Camus

**Affiliations:** 1 Service de Réanimation, CH de St BRIEUC, 10, rue Marcel Proust, 22000 Saint-Brieuc, France; 2 Service de Réanimation Médicale CHU de Rennes, 2, rue Henri le Guilloux, 35000 Rennes, France; 3 Service de Réanimation, CH de Vannes, 20, bd Maurice Guillaudot, 56000 Vannes, France; 4 Service de Réanimation Chirugicale, CHU de Rennes, 2, rue Henri le Guilloux, 35000 Rennes, France; 5 Service de Réanimation, CH Bretagne SUD, LORIENT, 5 avenue de Choiseul, 56322 Lorient, France; 6 Service de Réanimation, Centre Hospitalier de Saint-Malo, 1 rue de la Marne, 35400 Saint-Malo, France; 7 Service de Réanimation, Centre Hospitalier de Morlaix, 15 rue de kersaint gilly, 29600 Morlaix, France; 8 Service de Réanimation Médicale CHU de Brest, 2 avenue Foch, 29200 Brest, France; 9 Service de Réanimation, CH de QUIMPER, 14bis Avenue Yves Thépot, 29107 Quimper , France; 10 Service de Médecine Intensive Réanimation, CH de Saint-Nazaire, 11 bd Georges Charpak, 44600 Saint-Nazaire, France; 11 Service de Réanimation médicale, CHU de nantes, 1 place Alexis Ricordeau, 44093 Nantes , France

**Keywords:** Critical care, Pneumonia, Bacteremia, Mortality, COVID-19, Selective digestive decontamination, Mupirocin, Chlorhexidine

## Abstract

**Background:**

Among strategies that aimed to prevent acquired infections (AIs), selective decontamination regimens have been poorly studied in the COVID-19 setting. We assessed the impact of a multiple-site decontamination (MSD) regimen on the incidence of bloodstream infections (BSI) and ventilator-associated pneumonia (VAP) in COVID-19 patients receiving mechanical ventilation.

**Methods:**

We performed an ancillary analysis of a multicenter retrospective observational study in 15 ICUs in western France. In addition to standard-care (SC), 3 ICUs used MSD, a variant of selective digestive decontamination, which consists of the administration of topical antibiotics four times daily in the oropharynx and the gastric tube, chlorhexidine body wash and a 5-day nasal mupirocin course. AIs were compared between the 3 ICUs using MSD (MSD group) and the 12 ICUs using SC.

**Results:**

During study period, 614 of 1158 COVID-19 patients admitted in our ICU were intubated for at least 48 h. Due to missing data in 153 patients, 461 patients were finally included of whom 89 received MSD. There were 34 AIs in the MSD group (2117 patient-days), as compared with 274 AIs in the SC group (8957 patient-days) (*p* < 0.001). MSD was independently associated with a lower risk of AI (IRR = 0.56 [0.38–0.83]; *p* = 0.004) (Table 2). When the same model was used for each site of infection, MSD remained independently associated with a lower risk of VAP (IRR = 0.52 [0.33–0.89]; *p* = 0.005) but not of BSI (IRR = 0.58, [0.25–1.34], *p* = 0.21). Hospital mortality was lower in the MSD group (16.9% vs 30.1%, *p* = 0.017).

**Conclusions:**

In ventilated COVID-19 patients, MSD was independently associated with lower AI incidence.

**Supplementary Information:**

The online version contains supplementary material available at 10.1186/s13613-022-01057-x.

## Background

Despite increased knowledge regarding severe acute respiratory syndrome coronavirus 2 (SARS-CoV-2) epidemic, intensivists have to face a surge of critically ill patients worldwide and mechanical ventilation remained an inescapable lifesaving therapy. As it has been commonly described for other critically ill patients, those admitted in ICU with SARS-CoV-2 infectious disease (COVID-19) are at high risk for developing ventilator-associated infection (VAP) [1–3] and bloodstream infection (BSI) [4, 5] with implications for outcome. For four decades now, various selective oropharyngeal/digestive decontamination regimens have been reported to decrease the incidence of VAP, BSI and to some extent mortality [6–8] without increasing the risk of in multi-drug resistant (MDR) bacteria acquisition. To our knowledge, selective digestive decontamination has been rarely studied in the COVID-19 setting [9–11]. Of note, two studies reported survival benefit [10, 11]. Therefore, we conducted an observational study to assess the impact of a selective digestive decontamination regimen on acquired infections and survival in ICU COVID-19 patients. We hypothesized that this strategy could be associated with a reduction of the incidence of VAP and BSI but also with a reduced mortality rate.

## Methods

### Setting and patients

We performed a retrospective analysis mostly using the COCOREVAP cohort patients and some more patients of the Vannes and Saint–Brieuc centers. The COCOREVAP cohort is a multicenter retrospective observational study in 15 ICUs from 11 centers in western France. All adults admitted with COVID-19 from February 1st 2020 until December 31th 2021 who required mechanical ventilation were eligible. Additional patients in the Vannes and Saint–Brieuc centers were included between June the 1st and December 31th 2021. Patients under liberty deprivation (i.e., are under individual protection measure, such as tutorship and curatorship), pregnant women and patients younger than 18 years were excluded from the study. In addition to standard care (SC), three ICUs used a multiple-site decontamination regimen (MSD) for the prevention of acquired infections in intubated patients. MSD was used in all patients in one center (Rennes) and since May 5, 2021 in the two others (Saint–Brieuc and Vannes). Multiple-site decontamination is a variant of selective digestive decontamination, which consists of the administration of topical antibiotics including an aminoglycoside (tobramycin, 300 mg per day, in Rennes or gentamicin, 543 mg per day, in the two others centers), colistin sulfate (400 mg per day) and amphotericin B (2 g per day), four times daily in the oropharynx and the gastric tube, chlorhexidine body washing once daily and a 5-day nasal mupirocin course in patients who had an expected intubation duration of 24 h or more throughout the duration of intubation. Full details about the MSD regimen have been reported elsewhere [12]. Patients in the others ICUs received standard care alone. Patients who required intubation for an expected duration greater than 2 days were eligible for study and divided into two groups: MSD group and SC group.

Each center had a nosocomial infection committee for the prevention and prospective census of acquired infections and applied the recommendations of the French Society for Hospital Hygiene for the prevention and treatment of infection (available at https://sf2h.net/publications/actualisation-precautions-standard-2017).

The study protocol received approval from the ethical committee of the French Intensive Care Society (CE 21–56). Patients or closest relative were informed of the anonymous prospective collection of the data and had the possibility not to participate in the study. In case of refusal, the data were not collected accordingly. This manuscript follows the STROBE statement for reporting cohort studies.

### Definition

Infection was considered acquired in the ICU when it was diagnosed 48 h after admission and was not incubating on admission. BSI was defined as a positive blood culture occurring 48 h or more after admission. Regarding common skin contaminants, 2 positives blood cultures drawn on separate occasions were required [4]. The diagnosis of VAP was considered in patients ventilated for 48 h or more and was based on clinical signs (fever, purulent sputum, hypoxia), radiological findings (new infiltrate on chest-X-ray or CT scan), and leukocytosis [13]. Microorganisms responsible for infection were considered as multi-drug resistant (MDR) according to the European Society of Clinical Microbiology and Infectious Disease definition [14]. Respiratory samples used for VAP diagnosis were performed either with broncho-alveolar lavage, endotracheal aspiration or distally protected samples according with local protocols. To take into account the diagnosis heterogeneity of VAP among centers, the variable “Strategy for VAP diagnoses in center of admission” was created. It corresponds to the more frequently used pulmonary sample for VAP diagnosis in the center in which the patients was admitted. Of note, 7 centers mainly used endotracheal aspiration, 4 performed a majority of distally protected samples and the others performed broncho-alveolar lavage.

### Primary and secondary endpoints

The primary endpoint was the incidence of ICU-acquired infections, and secondary endpoints were specific VAP and BSI incidences as well as in hospital mortality.

### Statistical analysis

Statistical analysis was performed with the statistical software R 4.1.1. Categorical variables were expressed as percentages and continuous variables as median and interquartile range (IQR). The chi-square test and Fisher exact test were used to compare categorical variables and the Mann–Whitney *U* test or the Wilcoxon for continuous variables. Overall, 6.1% of the data were missing (192 patients had at least one missing data). For the purpose of the multivariable analysis, missing data were considered as missing at random and were handled using chained equation, using “MICE” R package to create an imputed data set.

Incidence rate and risk factors for acquired infections were analyzed using a univariate and multivariable Poisson regression model. Survival analysis were conducted with Kaplan–Meier survival curves with log-rank test and logistic regression with a stepwise backward regression using Akaike criteria as a stopping rule. Non-redundant variables associated with event (acquired infection or death) with a *p* value < 0.2 in the univariate analysis were included in the multivariable analysis.

To draw unbiased marginal estimates of exposure effect, a propensity-score matched analysis was performed. Propensity score was calculated using a non-parsimonious model (including all available baseline characteristics: age, male sex, body mass index, comorbidities, period of admission, inter-hospital transport, localization before admission, simplified acute physiology score II, bacterial co-infection at admission, biological parameters at admission, strategy for VAP diagnoses in center of admission and early management) and correspond for each patient to his probability to be admitted in an ICU, where MSD is implemented. Because of the non-parsimonious design, interaction effects between variables were not taken into account (e.g., between age and SAPS II scores). Using the “MatchIt” package, a k-nearest neighbor algorithm was used for propensity-score matching with a 1:1 ratio. The balance between matched groups was evaluated by the analysis of the standardized mean differences after weighting.

All tests were two-sided, and *p* < 0.05 was considered statistically significant.

## Results

### Population

**Fig. 1:**
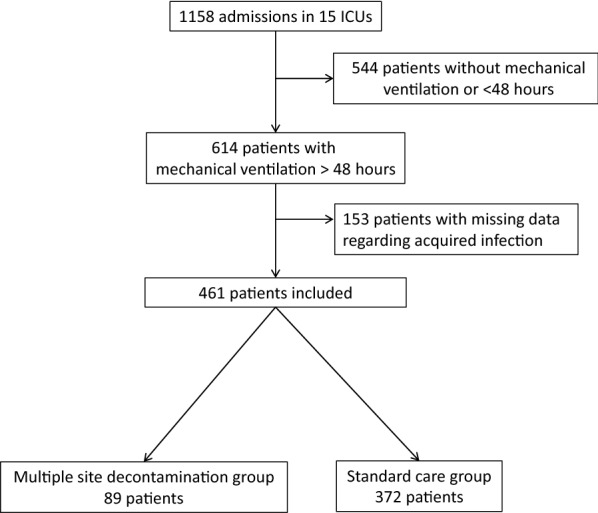
Flow chart

During the study period, 1158 patients were admitted in our ICUs with a COVID-19 diagnosis confirmed with a PCR, of whom 614 were intubated for at least 48 h. Due to missing data regarding AIs in 153 patients, 461 patients were included in the final study, of whom 89 received MSD (Fig. 1). Data regarding excluded patients are available in Supplementary materials. At admission, the simplified acute physiology score II was 36 [29–45], PaO2/FiO2 ratio was 100 [70–145] and 30.0% of patients were transferred from another ICU. Most baseline characteristics of MSD and SC patients were similar except a lower age (62 years [55–71] vs 68 [61–73], *p* < 0.05), a lower body mass index (27.46 [24.39–31.40] vs 28.76 [25.38–32.16], *p* < 0.05), a lower proportion of patients admitted during fall 2020 (31.5% vs 48.1%, *p* = 0.001) and a higher proportion of patients receiving antibiotic at admission (93.2% vs 82.3%, *p* = 0.018) in the MSD group (Table 1).

**Table 1:** Baseline characteristics and outcomes of study patients

Variables	Missing values	Standard care group	Multiple site decontamination group	*p* value
		*n* = 372	*n* = 89	
Age, year	0	68 [61–73]	62 [55–71]	0.002
Male—no. (%)	0	279 (75.0)	62 (69.7)	0.370
Body masse index, kg/m^2^	0	28.76 [25.38–32.16]	27.46 [24.39–31.40]	0.026
Comorbidities				
Chronic heart failure—no. (%)	0	60 (16.1)	13 (14.6)	0.848
Chronic respiratory disease—no. (%)	0	76 (20.4)	11 (12.4)	0.110
Chronic renal failure—no. (%)	0	35 (9.4)	5 (5.6)	0.352
Cirrhosis—no. (%)	0	16 (4.3)	7 (7.9)	0.264
Cancer—no. (%)	0	53 (14.2)	12 (13.5)	0.987
Immunodepression—no. (%)	0	57 (15.3)	9 (10.1)	0.275
Diabetes—no. (%)	0	105 (28.2)	24 (27.0)	0.915
Hypertension—no. (%)	0	186 (50.0)	41 (46.1)	0.583
Period of admission	0			< 0.001
Spring–Summer 2020—no. (%)		171 (46.0)	39 (43.8)	
Fall–Winter 2020—no. (%)		179 (48.1)	28 (31.5)	
Spring–Summer 2021—no. (%)		6 (1.6)	10 (11.2)	
Fall–Winter 2021—no. (%)		16 (4.3)	12 (13.5)	
Inter-hospital transport—no. (%)	0	114 (30.6)	24 (27.0)	0.581
Localization before ICU admission	0			0.408
Emergency ward—no. (%)		160 (43.0)	44 (49.4)	
Acute care ward—no. (%)		183 (49.2)	42 (47.2)	
Home—no. (%)		23 (6.2)	2 (2.2)	
Simplified acute physiology score II	46	37 [30–45]	35 [27–45]	0.334
Bacterial co-infection at admission—no. (%)	7	30 (8.2)	9 (10.2)	0.690
Biological parameters at admission				
Leucocytes × 10^9^/L	54	8.40 [6.17–10.96]	9.20 [6.15–10.95]	0.741
Lymphocytes × 10^9^/L	109	0.72 [0.52–1.00]	0.62 [0.46–0.87]	0.111
Platelets × 10^9^/L	51	217.00 [162.00–270.50]	213.00 [167.50–288.50]	0.475
Creatinine, µg/L	53	78.00 [65.00–106.00]	78.50 [63.25–101.00]	0.681
Serum C-reactive protein, mg/mL	192	140.00 [83.00–226.00]	139.50 [100.75–245.00]	0.625
Fibrinogene, g/L	173	6.70 [5.61–7.76]	6.90 [6.02–7.90]	0.334
PaO2/FiO2 ratio, mmHg	65	100.00 [65.00–135.85]	100.00 [80.00–157.78]	0.088
Early management				
High flow oxygenation before intubation—no. (%)	0	206 (55.4)	46 (51.7)	0.610
Systemic antibiotic at admission—no. (%)	6	302 (82.3)	82 (93.2)	0.018
Time from hospital admission to intubation, days	0	0.00 [0.00–1.00]	0.00 [0.00–2.00]	0.967
Antiviral agents—no. (%)	7	107 (29.2)	33 (37.5)	0.168
Steroids—no. (%)	7	256 (68.8)	63 (70.8)	0.82
Outcomes				
ICU acquired infection– no. (%)	0	220 (59.1)	29 (32.5)	< 0.001
Ventilator-associated pneumonia—no. (%)	0	212 (57.0)	26 (29.2)	< 0.001
Bloodstream infection—no. (%)	0	48 (12.9)	8 (9.0)	0.404
Length of mechanical ventilation, days	0	15.00 [8.00–26.00]	16.00 [8.50–24.00]	0.971
Length of stay, days	0	19.00 [12.00–30.25]	20.00 [12.00–30.00]	0.928
Hospital death—no. (%)	0	112 (30.1)	15 (16.9)	0.017

### Acquired infections

In the MSD group, compared with the SC group, there were 34 AIs (26 VAP, 8 BSI) in 2117 patients-days and 274 AIs (212 VAP, 62 BSI) in 8957 patients-days, respectively (incidence rate ratio [IRR] = 0.53, 95% CI 0.37–0.75, *p* < 0.001) (Table 1). Similarly, the VAP incidence rates were 14.3 per 1000 ventilatory-days and 28.3 per 1000 ventilatory-days, respectively (IRR = 0.51 [0.34–0.76], *p* < 0.001). There were numerically less BSI in the MSD group, with incidence rates of 3.79 and 6.9 per 1000 patients-days, respectively (IRR = 0.55, [0.26–1.14]; p = 0.10). In a multivariable Poisson regression model, MSD administration was associated with a lower risk of AI (IRR = 0.56 [0.38–0.83]; p = 0.004) (Table 2). When the same model was used for each site of infection, MSD remained independently associated with a lower risk of VAP (IRR = 0.52 [0.33–0.89]; p = 0.005) but not with a lower risk of BSI (IRR = 0.58, [0.25–1.34], p = 0.21) (not shown). Other risk factors for AI were male sex (IRR = 1.38 [1.03–1.86] p = 0.034), cirrhosis (IRR = 1.81 [1.17–2.82] p = 0.007), inter-hospital transport (IRR = 1.31 [1.02–1.69] p = 0.038) and higher simplified acute physiology score II (IRR = 1.01 per supplementary point [1.00–1.02] p = 0.032).

**Table 2:** Risk factors for ICU acquired infection

Variables	Univariate		Multivariable			
	IRR	95%CI	*p* value	IRR	95%CI	*p* value
Multiple site decontamination	0.50	0.35–0.72	< 0.001	0.56	0.38–0.83	0.004
Age, per supplementary year	1.01	1.00–1.02	0.086	1.01	0.99–1.02	0.28
Male	1.23	0.93–1.62	0.151	1.38	1.03–1.86	0.034
BMI, per supplementary kg/m^2^	1.00	0.98–1.02	0.70			
Comorbidities						
Chronic heart failure	0.96	0.71–1.29	0.77			
Chronic respiratory disease	1.01	0.77–1.33	0.93			
Chronic renal failure	1.37	0.97–1.93	0.075	1.21	0.81–1.81	0.34
Cirrhosis	1.64	1.08–2.48	0.021	1.81	1.17–2.82	0.007
Cancer	1.06	0.87–1.43	0.72			
Immunodepression	0.96	0.70–1.30	0.79			
Diabetes	1.00	0.78–1.28	0.99			
Hypertension	1.10	0.88–1.38	0.38			
Period of admission						
Spring–Summer 2020	Ref	Ref	Ref	Ref	Ref	Ref
Fall–Winter 2020	1.14	0.91–1.44	0.26	1.10	0.85–1.43	0.46
Spring–Summer 2021	0.53	0.22–1.30	0.165	0.81	0.32–2.06	0.66
Fall–Winter 2021	0.82	0.48–1.41	0.479	1.06	0.61–1.86	0.83
Inter-hospital transport	1.22	0.96–1.53	0.096	1.31	1.02–1.69	0.038
Localization before ICU admission						
Emergency ward	Ref	Ref	Ref			
Acute care ward	0.96	0.76–1.21	0.71			
Home	1.12	0.71–1.78	0.62			
Simplified acute physiology score II, per supplementary point	1.01	1.00–1.02	0.004	1.01	1.00–1.02	0.032
Bacterial co-infection at admission	1.20	0.82–1.75	0.36			
Biological parameters at admission						
Leucocytes per supplementary 10^9^/L	1.00	1.00–1.01	0.091	1.00	0.99–1.01	0.25
Lymphocytes per supplementary 10^9^/L	1.00	1.00–1.01	0.18			
Platelets per supplementary 10^9^/L	1.00	1.00–1.00	0.74			
Creatinine per supplementary µg/L	1.00	0.99–1.00	0.17	1.00	0.99–1.00	0.43
Serum C-reactive protein, per supplementary mg/mL	1.00	1.00–1.01	0.44			
Fibrinogene, per supplementary g/L	1.02	0.96–1.09	0.49			
PaO2/FiO2 ratio, per supplementary mmHg	1.00	0.99–1.00	0.19	0.99	0.99–1.01	0.31
Early management						
High flow oxygenation before intubation	1.01	0.81–1.27	0.90			
Systemic antibiotic at admission	0.80	0.60–1.06	0.13	0.87	0.63–1.19	0.39
Time from hospital admission to intubation, per supplementary days	0.96	0.91–1.01	0.088	0.97	0.91–1.01	0.11
Antiviral agents	0.89	0.69–1.14	0.36			
Steroids	1.14	0.88–1.48	0.33			
Strategy for VAP diagnoses in center of admission						
Endotracheal aspiration	Ref	Ref	Ref	Ref	Ref	Ref
Distally protected sample	1.13	0.90–1.43	0.29	1.02	0.79–1.32	0.89
Broncho alveolar lavage	1.47	0.95–2.27	0.087	1.45	0.90–2.33	0.12

Poisson regression model

### Microorganisms responsible for infection

Microorganisms responsible for infection are reported in Table 3. There were no differences between groups regarding either the responsible microorganisms or clinical presentation at VAP onset. There were 5 and 43 infections due to MDR microorganisms in the MSD and the SC group, respectively (*p* = 0.765).

**Table 3:** Characteristics of ICU acquired infections

Variables		Standard care group	Multiple site decontamination group	*p* value
	Missing value	*n* = 274	*n* = 34	
Time from admission to first AI, days	18	8.00 [5.00–11.50]	9.00 [5.00–15.00]	0.284
Ventilator associated pneumonia—no. (%)	0	*n* = 212	*n* = 26	1
Microorganism responsible for VAP*	0			
Non-fermenting Gram Negativ bacilli—no. (%)		43 (20.3)	8 (30.8)	0.329
Enterobacteriaceae—no. (%)		69 (32.5)	11 (42.3)	0.439
Staphylococcus aureus—no. (%)		46 (21.7)	1 (3.8)	0.034
Other Gram positive cocci—no. (%)		22 (10.4)	2 (7.7)	1.000
Enterococcus sp—no. (%)		4 (1.9)	2 (7.7)	0.131
Others—no. (%)		71 (33.5)	7 (26.9)	0.651
Multi-drug resistant microorganisms—no. (%)	82	32 (23.5)	5 (25.0)	1.000
Presentation at first ventilator-associated pneumonia				
Temperature on the day of diagnosis, °C	94	38.50 [38.00–39.00]	38.85 [37.25–39.18]	0.627
Leucocytes on the day of diagnosis × 10^9^/L	93	12.94 [9.88–17.21]	12.90 [10.35–15.50]	0.992
New radiological infiltrate—no. (%)	118	171 (78.4)	23 (82.1)	0.837
Bloodstream infection—no. (%)	0	*n* = 62	*n* = 8	1
Microorganism responsible for Bloodstream infection*	6			
Non-fermenting Gram Negativ bacilli—no. (%)		11 (19.6)	1 (12.5)	1
Enterobacteriaceae—no. (%)		18 (32.1)	1 (12.5)	0.418
Staphylococcus aureus—no. (%)		13 (23.2)	2 (25.0)	1
Other Gram positive cocci—no. (%)		12 (21.4)	1 (12.5)	1
Enterococcus sp— no. (%)		7 (12.5)	3 (37.5)	0.102
Others—no. (%)		30 (53.6)	3 (37.5)	0.468
Multi-drug resistant microorganisms—no. (%)	6	11 (19.6)	0 (0.0)	0.332

^*^As some infections were polymicrobial, total number of micro-organisms identified as responsible for acquired infections can be higher than number of infections

### Outcomes

**Fig. 2:**
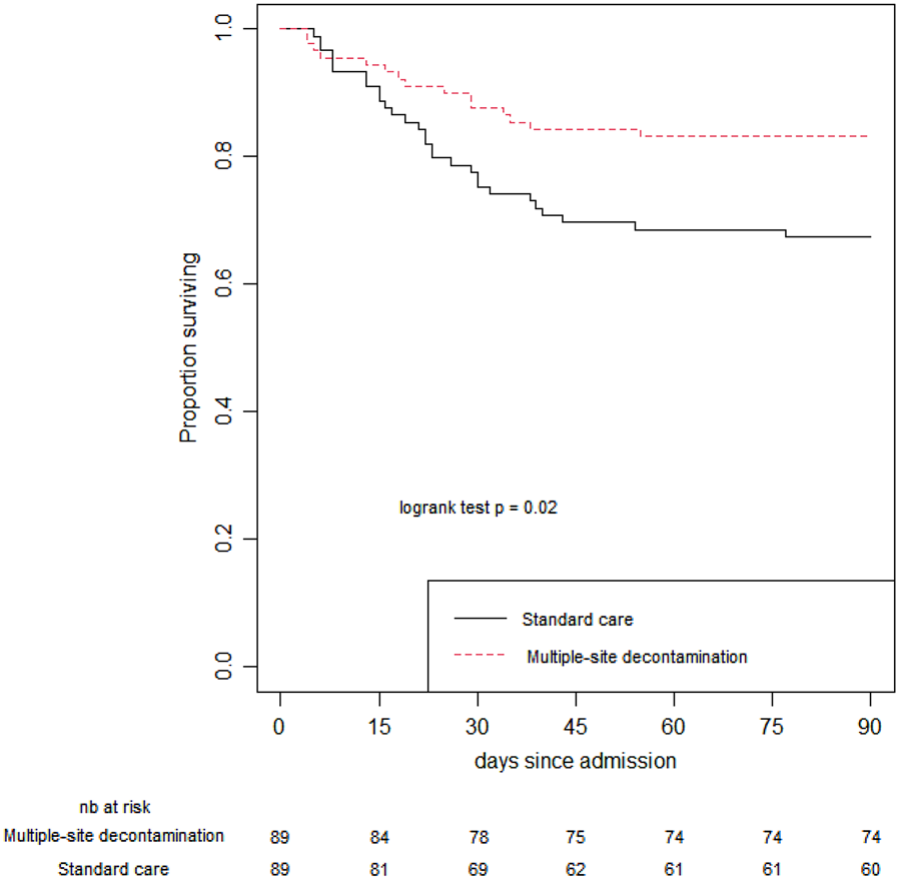
Survival curves in matched patient’s pairs

Fifteen patients (16.9%) in the MSD group died during hospital stay as compared with 112 patients (30.1%) in the SC group (p = 0.017; Table 1 and Fig. 2). In a multivariable logistic regression analysis, MSD remained independently associated with a lower risk for death (OR = 0.49 [0.24–0.99]; *p* = 0.049) (Additional file 1: Table S2). Others independent risk factors for in-hospital death were a higher age (OR = 1.06 per supplementary year, [1.03–1.09] *p* < 0.001), inter-hospital transport (0.40 [0.22–0.74] *p* = 0.03), higher SAPS II (OR = 1.05 per supplementary point, [1.03–1.07] *p* < 0.001) and systemic antibiotic at admission (OR = 0.47 [0.25–0.88] *p* = 0.018).

### Propensity score matched analysis

Then, patients who received MSD where matched with similar patients who received standard care using a non-parsimonious propensity score matching. Matching process resulted in 89 matched patient’s pairs with well-balanced baseline characteristics (Table 4). In this data set, patients of the MSD group still had a lower incidence of AI as compared patients receiving standard care (32.6% vs 57.3% *p* = 0.002), mainly because of a lower incidence of VAP (29.2% vs 53.9% *p* = 0.001), while BSI incidence was not statistically different (7.9% vs 11.2% *p* = 0.61). Finally, hospital mortality rate remained lower in the MSD group as compared with standard care group (16.9% vs 32.6% *p* = 0.024).

**Table 4:** Baseline characteristics and outcomes of matched patients pairs (propensity score matched analysis)

Variables	Standard care group	Multiple site decontamination group	*p* value	SMD
	*n* = 89	*n* = 89		
Age, year	66 [55–71]	62 [55–71]	0.469	0.094
Male,—no. (%)	66 (74.2)	62 (69.7)	0.617	0.097
Body masse index, kg/m^2^	28.28 [24.69–31.38]	27.46 [24.39–31.40]	0.623	0.090
Comorbidities				
Chronic heart failure—no. (%)	15 (16.9)	13 (14.6)	0.837	0.063
Chronic respiratory disease—no. (%)	15 (16.9)	11 (12.4)	0.524	0.137
Chronic renal failure—no. (%)	5 (5.6)	5 (5.6)	1.000	0.00
Cirrhosis—no. (%)	6 (6.7)	7 (7.9)	1.000	0.042
Cancer—no. (%)	11 (12.4)	12 (13.5)	1.000	0.033
Immunodepression—no. (%)	6 (6.7)	9 (10.1)	0.589	0.111
Diabetes—no. (%)	28 (31.5)	24 (27.0)	0.621	0.101
Hypertension—no. (%)	45 (50.6)	41 (46.1)	0.653	0.090
Period of admission			0.254	
Spring–Summer 2020—no. (%)	35 (39.3)	39 (43.8)		0.05
Fall–Winter 2020—no. (%)	37 (41.6)	28 (31.5)		0.10
Spring–Summer 2021—no. (%)	4 (4.5)	10 (11.2)		0.07
Fall–Winter 2021—no. (%)	13 (14.6)	12 (13.5)		0.01
Inter-hospital transport—no. (%)	34 (38.2)	24 (27.0)	0.150	0.253
Localization before ICU admission			0.919	
Emergency ward—no. (%)	47 (52.8)	44 (49.4)		0.03
Acute care ward– no. (%)	38 (42.7)	42 (47.2)		0.05
Home—no. (%)	3 (3.4)	2 (2.2)		0.01
Simplified acute physiology score II	34 [27–43]	35 [27–45]	0.729	0.063
Bacterial co-infection at admission—no. (%)	6 (6.7)	9 (10.1)	0.589	0.112
Biological parameters at admission				
Leucocytes × 10^9^/L	8.10 [6.23–10.40]	9.20 [6.10–11]	0.602	0.044
Lymphocytes × 10^9^/L	0.72 [0.50–1.02]	0.65 [0.47–0.90]	0.273	0.078
Platelets × 10^9^/L	210 [165–259]	214 [169–293]	0.248	0.232
Creatinine, µg/L	76 [61.60–101]	79 [63—103]	0.643	0.105
Serum C-reactive protein, mg/mL	119 [72–234]	139 [90–228]	0.307	0.125
Fibrinogene, g/L	6.60 [5.60–7.43]	7.14 [6.12–8]	0.19	0.22
PaO2/FiO2 ratio, mmHg	100 [70–133.03]	100 [80–161.90]	0.291	0.098
Strategy for VAP diagnoses in center of admission				
Endotracheal aspiration	77 (86.5)	77 (86.5)	1.00	0.00
Distally protected sample	12 (13.5)	12 (13.5)	1.00	0.00
Broncho alveolar lavage	0	0	–	0.00
Early management				
High flow oxygenation before intubation—no. (%)	45 (50.6)	46 (51.7)	1.000	0.022
Systemic antibiotic at admission—no. (%)	80 (89.9)	83 (93.3)	0.589	0.134
Time from hospital admission to intubation, days	0 [0–2]	0 [0–2]	0.937	0.119
Antiviral agents—no. (%)	35 (39.3)	33 (37.1)	0.877	0.047
Steroids—no. (%)	59 (66.3)	63 (70.8)	0.628	0.099
Outcomes				
ICU acquired infection—no. (%)	51 (57.3)	29 (32.6)	0.002	
Ventilator-associated pneumonia—no. (%)	48 (53.9)	26 (29.2)	0.001	
Bloodstream infection—no. (%)	10 (11.2)	7 (7.9)	0.610	
Length of mechanical ventilation, days	15 [8–26]	16 [8–24]	0.791	
Length of stay, days	19 [10–30]	20 [12–30]	0.761	
Hospital death—no. (%)	29 (32.6)	15 (16.9)	0.024	

*SMD* Standardized mean difference

## Discussion

In this observational study conducted in ICUs with low multi-drug resistance rate [15], we have shown that MSD is associated with a decreased risk of AI but also with a better survival in COVID-19 critically ill patients. To our knowledge, only one study suggested a survival benefit with selective decontamination regimen in this setting [10], but our study is the first that specifically investigate relationship between selective digestive decontamination regimen and AI.

Prolonged mechanical ventilation, immunosuppressive treatments (i.e., corticosteroids and/or IL-6 signaling blockade) [16, 17] and immune dysfunctions [18] observed in severe COVID-19 patients might be responsible for their higher risk of nosocomial infections [1, 3, 5]. Moreover, viral pneumonia may induce damages to the ciliated cells, leading to impaired mucociliary clearance and increased risk for bacterial adhesion and colonization of the airways. While there is still a controversy on the degree of VAP attributable mortality, the occurrence of VAP seems to have a significant impact on the outcome of COVID-19 patients [3, 19] making the evaluation of preventive effective therapies a priority. Of note, the high incidence of VAP in our control group (57%) corresponds with the high incidence reported in the COVID–ICU study (58%) [1]. However, BSI incidence in the present study (12.5%) was lower than previously observed (19.5% to 29.6%) [5, 21] and our study might be underpowered to explore this specific site of AI.

Until now, attributable mortality of AI was mainly recognized for surgical patients but not for medical ones [20]. In contrast, Rouze et al. observed that VAP onset was independently associated with a poorer outcome in COVID-19 patients, whereas patients infected with other viruses had similar outcomes whether they had VAP or not [3]. This result supports a potential benefit for infection prevention strategy in this particular setting. Although, its benefit was not confirmed in study conducted in area with higher resistance rate [22], selective digestive decontamination regimens have been associated with reduced mortality and lower BSI and VAP rates in areas with low levels of antibiotic resistance. A major concern regarding decontamination regimen is the application of systematic antibiotic at admission. It is noteworthy that de Jonge et al. showed that selective digestive decontamination (including 4-day intravenous cefotaxime) was associated with higher levels of antibiotic susceptibility of Gram negative bacteria to ceftazidime, ciprofloxacin, imipenem and tobramycin [23]. Assuming a proportion of patients with a bacterial infection on admission between 10 and 25%, the systematic use of short-term antibiotic therapy to treat incubating pneumonia prevent early VAP seems reasonable [24, 25]. Finally, when analyzing the bacteria recovered from respiratory samples, the proportion of MDR bacteria was low and not statistically different between MSD and standard care groups.

To our knowledge, our study is the largest to evaluate the effects of selective digestive decontamination on ICU acquired infections in SARS-CoV-2 patients. Nevertheless, some limitations should be pointed out. First, our study was conducted on adult ICU in the west of France only, where MDR are not endemic. Hence, the effect of MSD observed here might not be generalizable to the whole population of COVID-19 ventilated patients. Second, as mentioned before, our study was retrospective that implies missing data. Third, due to a marked disproportion between groups with a relatively small sample size in the MSD group, our study might not be adequately powered to detect some differences between groups (such as differences in age or severity score). Fourth, VAP is a subjective endpoint and physicians may have been influenced in their diagnosis because of the non-blind nature of the study. Finally, other residual confounders, such as COVID-19-associated ICU surge, ARDS management but also heterogeneity between center for AI diagnosis and prevention were not assessed in our study.

To conclude, the incidence of AIs, especially VAP, was significantly lower in patients receiving a multiple-site decontamination regimen without significant impact on MDR bacteria acquisition.

## Supplementary Information

**Additional file 1: Table S1.** Baseline characteristics and outcomes of patients included and not included. **Table S2.** Risk factors for hospital death. **Figure S1.** Survival curves in patients receiving MSD or not in whole population (inclusion of the 153 patients with missing data regarding AI).

## Data Availability

The data sets generated during the current study are available from the corresponding author on reasonable request.
