# Supplementary material for: Multiple-site decontamination regimen decreases acquired infection incidence in mechanically ventilated COVID-19 patients
Source: Ann Intensive Care. 2022 Sep 2;12:84. doi: 10.1186/s13613-022-01057-x (PMC9438389; doi:10.1186/s13613-022-01057-x)

Additional file 1: Table S1. Baseline characteristics and outcomes of patients included and not included.

| Variables | Not included  n = 153 | Included  n = 461 | p-value |
| --- | --- | --- | --- |
| Multiple site decontamination | 20 (13.2) | 89 (19.3) | 0.110 |
| Age, year | 65.00 [52.00, 72.00] | 67.00 [59.00, 72.00] | 0.096 |
| Simplified acute physiology score II | 33 [27 – 41] | 36 [29 – 45] | 0.043 |
| Male, – no. (%) | 102 (67.1) | 341 (74.0) | 0.125 |
| Inter-hospital transport – no. (%) | 21 (13.8) | 138 (29.9) | <0.001 |
| Body masse index, kg/m² | 28.00 [25.00, 32.00] | 28.41 [25.06, 32.00] | 0.420 |
| Chronic heart failure– no. (%) | 26 (17.1) | 73 (15.8) | 0.809 |
| Chronic respiratory disease– no. (%) | 21 (13.8) | 87 (18.9) | 0.195 |
| Chronic renal failure– no. (%) | 9 (5.9) | 40 (8.7) | 0.361 |
| Cirrhosis– no. (%) | 3 (2.0) | 23 (5.0) | 0.171 |
| Cancer– no. (%) | 4 (2.6) | 65 (14.1) | <0.001 |
| Immunodepression– no. (%) | 17 (11.2) | 66 (14.3) | 0.400 |
| Diabetes– no. (%) | 36 (23.7) | 129 (28.0) | 0.352 |
| Hypertension– no. (%) | 56 (36.8) | 227 (49.2) | 0.010 |
| Time from hospital admission to intubation, days | 0.00 [0.00, 2.00] | 0.00 [0.00, 1.00] | 0.634 |
| Length of mechanical ventilation, days | 10.00 [2.50, 22.50] | 15.00 [8.00, 26.00] | <0.001 |
| Hospital death– no. (%) | 34 (22.4) | 127 (27.5) | 0.249 |

Additonal file 1: Table S2. Risk factors for hospital death.

| Variables | Univariate | | | Multivariable | |  |
| --- | --- | --- | --- | --- | --- | --- |
|  | OR | 95%CI |  | OR | 95%CI |  |
| Multiple site decontamination | 0.47 | 0.26-0.86 | 0.013 | 0.49 | 0.24-0.99 | 0.049 |
| Age, per supplementary year | 1.08 | 1.05-1.11 | <0.001 | 1.06 | 1.03-1.09 | <0.001 |
| Male | 1.43 | 0.88-2.33 | 0.15 | 1.66 | 0.93-2.94 | 0.085 |
| BMI, per supplementary kg/m² | 0.98 | 0.94-1.02 |  |  |  |  |
| *Chronic heart failure* | 1.36 | 0.79-2.32 | 0.27 |  |  |  |
| *Chronic respiratory disease* | 1.6 | 0.98-2.63 | 0.62 |  |  |  |
| *Chronic renal failure* | 2.09 | 1.08-4.06 | 0.029 |  |  |  |
| *Cirrhosis* | 0.92 | 0.36-2.4 | 0.872 |  |  |  |
| *Cancer* | 2.64 | 1.54-4.53 | <0.001 | 1.64 | 0.88-3.05 | 0.12 |
| *Immunodepression* | 1.75 | 1.01-3.02 | 0.044 |  |  |  |
| *Diabetes* | 1.03 | 0.65-1.61 | 0.915 |  |  |  |
| *Hypertension* | 1.51 | 1-2.28 | 0.049 |  |  |  |
| Period of admission |  |  |  |  |  |  |
| *Spring-Summer 2020* | Ref | Ref | Ref |  |  |  |
| *Fall-Winter 2020* | 1.72 | 1.12-2.65 | 0.013 |  |  |  |
| *Spring-Summer 2021* | 1.12 | 0.34-3.64 | 0.84 |  |  |  |
| *Fall-Winter 2021* | 0.73 | 0.26-2.03 | 0.55 |  |  |  |
| Inter-hospital transport | 0.32 | 0.19-0.54 | <0.001 | 0.40 | 0.22-0.74 | 0.003 |
| Localisation before ICU admission |  |  |  |  |  |  |
| *Emergency ward* | Ref | Ref | Ref |  |  |  |
| *Acute care ward* | 1.52 | 0.98-2.34 | 0.71 |  |  |  |
| *Home* | 2.29 | 0.96-5.44 | 0.060 |  |  |  |
| Simplified acute physiology score II *per supplementary point* | 1.05 | 1.03-1.07 | <0.001 | 1.05 | 1.03-1.07 | <0.001 |
| Bacterial co-infection at admission | 1.95 | 0.99-3.83 | 0.0518. | 1.42 | 0.64-3.13 | 0.38 |
| Biological parameters at admission |  |  |  |  |  |  |
| *Leucocytes per supplementary 10^9^/L* | 1.02 | 0.98-1.07 | 0.269 |  |  |  |
| *Lymphocytes per supplementary 10^9^/L* | 1.02 | 0.98-1.05 | 0.344 |  |  |  |
| *Platelets per supplementary 10^9^/L* | 1 | 0.99-1 | 0.023 | 1.00 | 0.99-1.00 | 0.055 |
| *Creatinine per supplementary µg/L* | 1 | 1-1 | 0.063 | 1.00 | 0.99-1.00 | 0.28 |
| *Serum C-reactive protein, per supplementary mg/mL* | 1 | 1-1 | 0.30 |  |  |  |
| *Fibrinogene, per supplementary g/L* | 0.88 | 0.76-1.02 | 0.0821. |  |  |  |
| *PaO2/FiO2 ratio, per supplementary mmHg* | 1 | 1-1 | 0.39 |  |  |  |
| Early management |  |  |  |  |  |  |
| *High flow oxygenation before intubation* | 1.4 | 0.92-2.12 | 0.113 |  |  |  |
| *Systemic antibiotic at admission* | 0.46 | 0.27-0.78 | 0.004 | 0.47 | 0.25-0.88 | 0.018 |
| *Time from hospital admission to intubation, per supplementary day* | 1.09 | 1.01-1.17 | 0.022 | 1.09 | 0.99-1.19 | 0.064 |
| *Antiviral agents* | 0.79 | 0.5-1.24 | 0.302 |  |  |  |
| *Steroids* | 2.18 | 1.33-3.57 | 0.002 | 1.73 | 0.98-3.07 | 0.060 |

Note. Logistic regression, multivariable model with multiple imputation and stepwise backward elimination using Akaike criteria

Additional file 1: Figure S1. Survival curves in patients receiving MSD or not in whole populaiton (inclusion of the 153 patients with missing data regarding AI).
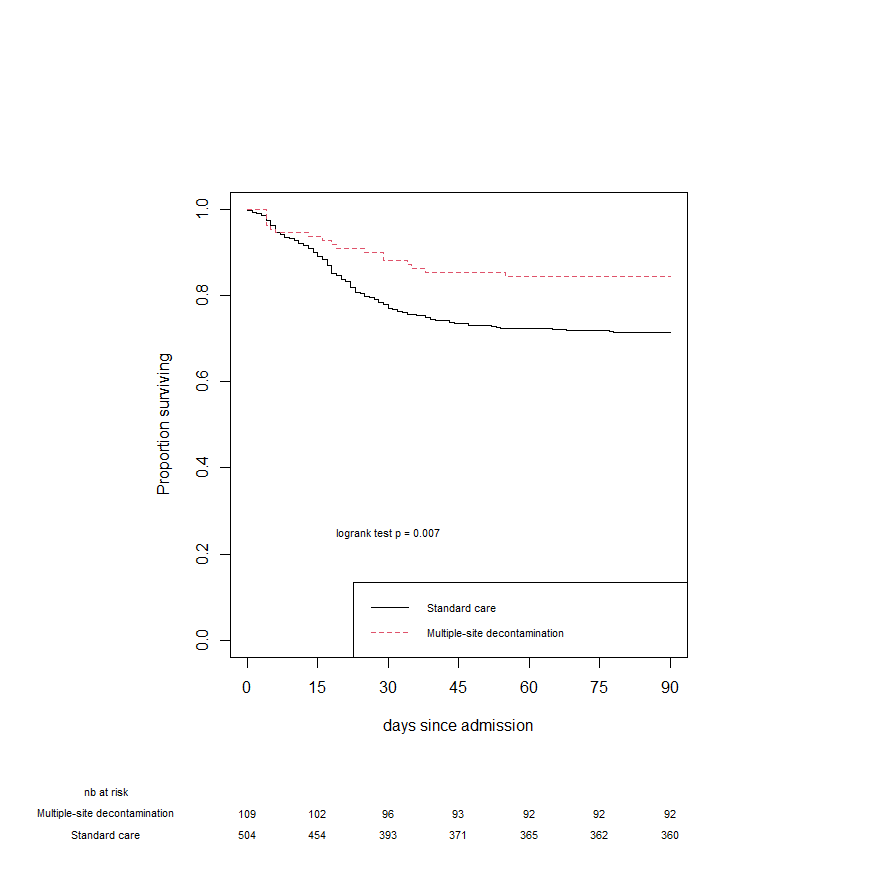

Supplement: Supplementary file 1 — Additional file 1: Table S1. Baseline characteristics and outcomes of patients included and not included. Table S2. Risk factors for hospital death. Figure S1. Survival curves in patients receiving MSD or not in whole population (inclusion of the 153 patients with missing data regarding AI). [file 13613_2022_1057_MOESM1_ESM.doc]
